# Smart Biomaterials in Wound Healing: Advances, Challenges, and Future Directions in Intelligent Dressing Design

**DOI:** 10.3390/bioengineering12111178

**Published:** 2025-10-29

**Authors:** Yanlin Liu, Liqin Ge

**Affiliations:** 1 State Key Laboratory of Digital Medical Engineering, School of Biological Science and Medical Engineering, Southeast University, Nanjing 210096, China; isliuyanlin@163.com; 2 Advanced Ocean Institute of Southeast University, Nantong 226000, China

**Keywords:** chronic wound healing, intelligent wound dressings, stimuli-responsive materials, biosensors

## Abstract

Chronic wounds (such as diabetic foot ulcers and pressure ulcers) affect millions of patients worldwide. These non-healing wounds pose major clinical challenges due to persistent inflammation, high infection risk, and impaired tissue regeneration, and incur a substantial healthcare burden, with global wound care costs reaching tens of billions of dollars annually. This unmet need has spurred the development of intelligent wound dressings—advanced bioengineered systems that go beyond conventional passive wound coverings by actively monitoring the wound microenvironment and responding dynamically to promote tissue repair. This review comprehensively examines a broad range of smart wound dressing technologies, including pH-sensitive, temperature-responsive, moisture-responsive, pressure-sensing, electroactive, biosensor-integrated, shape-memory, and controlled drug-releasing systems. We also discuss critical challenges in translating these innovations to clinical practice, such as ensuring biocompatibility and long-term stability in the harsh wound environment, manufacturing scalability and cost-effectiveness, patient comfort and adherence, and navigating regulatory hurdles. By emphasizing recent bioengineering advances and clinical potential, we underscore that intelligent wound dressings represent a paradigm shift in chronic wound management—enabling continuous, personalized therapy with the potential to significantly improve healing outcomes, reduce complications, and improve patient quality of life.

## 1. Introduction

Chronic wounds, such as diabetic foot ulcers, pressure ulcers, and venous ulcers, are a significant clinical challenge characterized by prolonged inflammation and failure to progress through normal healing phases [1]. Unlike acute wounds which typically heal in weeks, chronic wounds can persist for months or years, often remaining in an inflammatory state with excessive proteases and bacterial burden [2]. These non-healing wounds impose severe health and economic burdens: patients with chronic wounds have a five-year survival rate of only ~70%, worse than many cancers, and treatment costs exceed $25–28 billion annually in the U.S. Key barriers to healing include persistent infection, ischemia, biofilm formation, imbalanced moisture, and repeated trauma from frequent dressing changes [3].

In diabetic patients, chronic wounds (especially diabetic foot ulcers) arise from a perfect storm of systemic and local factors. Peripheral neuropathy—present in roughly half of diabetic individuals—leads to loss of protective sensation, causing repetitive unnoticed injuries and pressure that exacerbate ulcers. Concurrently, diabetic microangiopathy and peripheral arterial disease result in ischemia and poor perfusion around the wound, depriving tissues of oxygen and nutrients [4]. Impaired angiogenesis in the wound bed further stalls granulation tissue formation: diabetes-related hypoxia and decreased angiogenic growth factors severely blunt new vessel formation. Hyperglycemia and metabolic dysfunction also compromise the immune response, with dysfunctions like reduced neutrophil activity and macrophages locked in a pro-inflammatory phenotype; this immune dysregulation permits persistent infections and prolonged inflammation. Moreover, chronic wounds often develop polymicrobial biofilms, wherein bacteria are encased in a protective matrix that blocks antibiotics and immune cells. It’s reported that up to ~60% of diabetic foot ulcers contain resilient biofilms, which sustain inflammation and further impair healing [2]. Together, these factors create a hostile wound microenvironment that resists closure, characterized by impaired angiogenesis, immune dysfunction, neuropathy, and biofilm formation, particularly in patients with diabetes [5].

Traditional wound dressings (gauze, simple bandages) mainly provide coverage and moisture control, but they do not actively monitor or respond to the wound environment [6]. As a result, clinicians must frequently remove dressings to assess wounds visually or via cultures, which disrupts healing tissue and increases infection risk [7]. There is a compelling need for more “intelligent” wound dressings that can sense wound conditions and dynamically respond to promote healing.

Intelligent (or “smart”) wound dressings are an emerging class of biomaterial systems endowed with sensing and responsive functionalities. Unlike passive dressings, intelligent dressings can monitor parameters such as pH, temperature, moisture, and biochemical markers in real time, and trigger therapeutic actions like drug release, electrical stimulation, or color change to signal problems [8]. By combining sensing, feedback, and therapy delivery, these smart systems aim to create an optimal healing environment continuously and address chronic wound challenges such as uncontrolled infection, prolonged inflammation, and inadequate healing signals [9]. For example, a smart dressing might detect an infection via pH increase and automatically release antimicrobials or alert the patient, intervening before the infection worsens [10]. Another might monitor wound moisture and electrically stimulate the tissue if healing stalls [11]. In essence, intelligent dressings strive to perform active wound management, reducing the need for frequent clinical inspection and enabling earlier, targeted interventions.

The concept of optimizing the wound environment is not new—in 1962, Winter’s seminal work demonstrated that maintaining a moist wound environment accelerates healing [5]. This insight led to modern moisture-retentive dressings (films, hydrocolloids, hydrogels) in the latter 20th century, a major advance over dry gauze [12]. By the 21st century, research began integrating bioactive agents (e.g., growth factors, antimicrobials) into dressings for enhanced healing [13]. The last decade has seen a rapid rise of “smart” wound care technologies, propelled by advances in biomaterials, flexible electronics, and biosensors [8].

In this literature review, we comprehensively examine intelligent dressings in chronic wound healing, covering major categories of smart dressings—pH-sensitive, temperature-responsive, moisture-responsive, pressure-sensing, controlled drug-releasing, electroactive (electrical stimulation), biosensor-integrated, and shape-memory dressings [4]. For each category, we discuss the mechanism of action, material composition, and sensing/response function, highlighting how they tackle specific chronic wound problems. We summarize key findings from recent research (primarily within the past 10 years, both preclinical and clinical) and provide critical analysis of their efficacy [14]. We also address current limitations of these technologies, including technical, regulatory, and commercialization hurdles, and outline future research directions toward smarter wound management. The aim is to provide an overview of the state-of-the-art in smart wound dressings and their role in revolutionizing chronic wound care [15].

## 2. From Passive to Smart: Evolution of Wound Dressing Technologies

A brief historical perspective puts today’s intelligent dressings into context. Ancient wound care practices relied on natural materials with antiseptic properties—e.g., Mesopotamians (c. 2500 BC) applied honey and resin, and Hippocrates (460–370 BC) recommended cleaning wounds with wine or vinegar [16]. These early dressings primarily aimed to protect the wound and prevent infection. Fast-forward to the 19th century, the introduction of germ theory and antiseptics (Lister’s carbolic acid) and later antibiotics dramatically improved infection control [17]. However, traditional dressings (cotton gauze, wool pads) tended to desiccate wounds. In 1962, George Winter’s experiments demonstrated that wounds heal faster in a moist environment, challenging the convention of “dry healing” [11]. This discovery spurred development of modern dressings in the late 20th century that maintain moisture and promote autolytic debridement—e.g., occlusive films, foams, hydrocolloids, alginates, and hydrogels [18]. These advanced dressings improved healing rates and patient comfort, and by the 21st century thousands of wound care products became available.

The concept of “smart” wound dressings emerged as researchers sought to go beyond passive functions. Early smart dressings in the 2000s and 2010s incorporated indicator functionalities. A notable example is pH-indicator dressings that change color in alkaline conditions (signaling infection) [19]. Simple biochemical sensors (like enzyme-based colorimetric patches) were also explored to detect infection-related metabolites. Around the same time, stimuli-responsive polymers entered the scene—materials that alter their properties (e.g., swell, shrink, or release payloads) in response to stimuli like temperature or pH. This allowed the creation of dressings that respond to the wound state, not just sense it. For instance, thermo-responsive hydrogel dressings were designed to gel or release drugs when the wound is feverish [20]. In the last decade, rapid advances in flexible electronics, nanotechnology, and biomaterials converged to enable truly intelligent wound dressings. Researchers have integrated miniaturized sensors (measuring temperature, moisture, pH, biomarkers), wireless communication (NFC/Bluetooth), and actuators (micro-heaters, drug delivery pumps, electrodes) on thin, flexible substrates that can be applied like bandages [11]. Pioneering examples include a wireless bandage that continuously monitors impedance and temperature and delivers on-demand electrical stimulation to accelerate healing [11], and multilayer “smart bandages” with microfluidic drug reservoirs that release antibiotics when sensors detect infection [21].

## 3. Classification of Smart Biomaterials

Smart Biomaterials can be categorized by their typical stimuli and mechanisms. As Table 1 highlights, intelligent dressings encompass a wide array of stimuli and functionalities. The following sections provide an in-depth review of each major category, focusing on how they work (mechanisms of action), what materials enable their smart behavior, and evidence of their performance.

### 3.1. pH-Sensitive Dressings

One hallmark of chronic wounds is an elevated alkaline pH in the wound microenvironment. Healthy skin is slightly acidic (pH about 4.5 to 6.5), which inhibits bacterial growth, and acute healing wounds tend to remain in this acidic range [22]. In chronic or infected wounds, however, bacterial metabolism and tissue degradation products can raise the pH to 7–9 [23]. This pH shift is both a diagnostic indicator of infection and a factor that can impair healing (since many enzymatic processes in healing prefer mildly acidic conditions). pH-sensitive dressings leverage this phenomenon either to signal the change or to actively respond to it.

**Figure 1:**
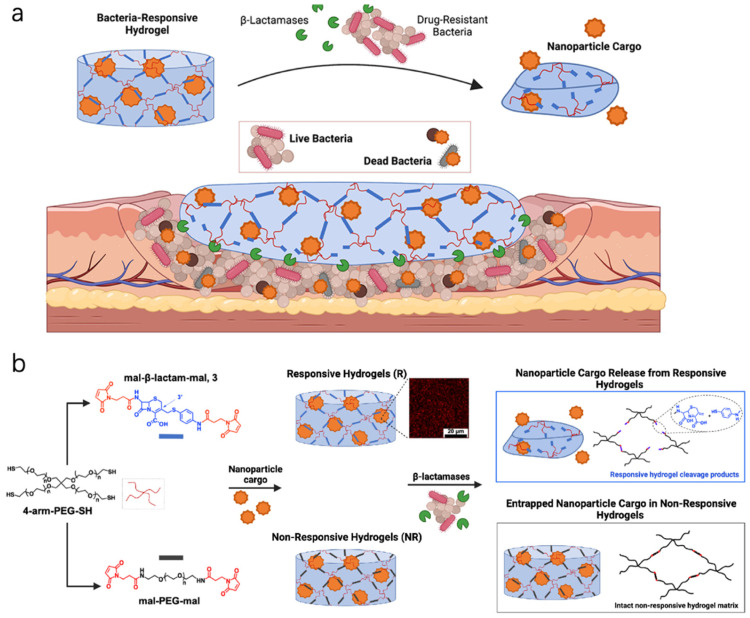
(**a**) β-Lactamase-Responsive Hydrogel Drug Delivery Platform for Bacteria-Triggered Cargo Release. (**b**) Schematic Representation of β-Lactamase-Responsive (R) Hydrogel Fabrication via Thiol−ene Michael-Type Addition. Reprinted from Ref. [26].

Colorimetric pH indicators are a simple yet effective approach. Dressings infused with pH-sensitive dyes can produce a visible color change when the wound becomes alkaline, alerting caregivers to possible infection without removing the dressing [24]. A classic example is bromothymol blue (BTB) integrated into a dressing: BTB is yellow at pH ~5–6 but turns blue above pH ~7. Researchers have loaded BTB into nanoporous silica particles within a nanocellulose mesh dressing. In an uninfected wound (pH ≈ 5.5), the dressing stays yellow, but if infection causes pH ≥ 8, the dressing turns blue within minutes [19]. This color change can be observed through a transparent secondary cover, providing an instant visual cue of infection onset (Figure 1a). In vitro, such BTB-nanocellulose dressings detected pH changes before clinical signs like pus or redness appeared, offering earlier intervention opportunities [19].

Natural pH-sensitive compounds have also been explored to avoid synthetic dye chemicals. For instance, anthocyanins from red cabbage or berries exhibit pH-dependent color changes. Arafa et al. developed a chitosan hydrogel dressing incorporating red cabbage extract; in simulations of wound fluid, the hydrogel shifted from purple to green as pH rose into the infection range [23]. These bio-based indicators are attractive for their biocompatibility.

Beyond passive indication, some pH-sensitive dressings are designed to respond therapeutically [25]. One approach is pH-triggered drug release. In an infected, alkaline wound, such a dressing could automatically release antimicrobials where and when needed, while keeping them sequestered at normal pH to avoid overuse. Shukla and colleagues created a hydrogel that remains intact in neutral pH but degrades in the presence of bacterial byproducts like β-alanine or enzymes that accompany high-bacterial-load (alkaline) conditions [26] (Figure 1a). Specifically, they used a polymer sensitive to β-lactamase (an enzyme secreted by many bacteria): when β-lactamase is present (indicating infection), it cleaves the polymer crosslinks, causing the hydrogel to dissolve and release encapsulated antibiotic nanoparticles. This is a clever inversion of bacterial resistance mechanisms—β-lactamase normally helps bacteria by destroying certain antibiotics, but here its presence triggers the dressing to unleash a drug payload against the bacteria [26] (Figure 1b). In lab tests, this system released virtually no drug in the absence of bacteria, but quickly delivered antibiotics upon contact with β-lactamase-producing bacteria, thereby targeting infection on demand [26].

The materials enabling pH responsiveness are often polymers with ionizable groups. Chitosan, for example, is a cationic biopolymer that swells or dissolves depending on pH (soluble in acidic medium, precipitates in alkaline). This makes it useful for fabricating films that might, say, remain intact on healthy skin (acidic) but soften in an infected wound (alkaline), potentially releasing loaded drugs. Other polymers like Eudragit or poly(L-glutamic acid) have been used to create pH-dependent coatings for drug reservoirs. pH-electrodes and potentiometric sensors represent another strategy: tiny pH sensors (like those used in lab pH meters) can be integrated into a dressing and connected to a readout device to give quantitative pH readings [27]. Flexible printed pH sensor strips have been demonstrated—this use conducting polymer electrodes modified to be sensitive to H^+^ concentrations and can continuously log the wound pH when left in place [28].

In summary, pH-sensitive intelligent dressings act as “infection sensors” and potentially “on-demand drug releasers” for chronic wounds. By taking advantage of the wound’s biochemical signature, they can provide earlier warnings of complications and deliver therapy more selectively [29]. Their appeal also lies in the straightforward clinical translation: pH is a well-known marker of wound status and pH indicator dyes are inexpensive and easy to interpret (a simple color change). This makes pH-responsive dressings one of the most practically viable smart dressing strategies—even resource-limited settings could potentially use a color-changing bandage to detect infection.

### 3.2. Temperature-Responsive Dressings

Temperature is a fundamental parameter reflecting a wound’s condition. A healing wound typically maintains a near-normal core temperature (about 37 °C), whereas deviations can signal trouble: increased local temperature often indicates infection or excessive inflammation, and a drop in temperature may signal poor blood flow or ischemia in the area [30]. Clinically, a sustained rise of even ~1–2 °C at a wound site can herald infection before other symptoms manifest [31]. Temperature-responsive wound dressings aim to monitor or respond to these changes to optimize healing conditions [32].

There are two main aspects to temperature-smart dressings: temperature sensing and temperature-triggered actuation [33]. On the sensing side, the simplest form is using thermochromic materials that change color with temperature [34]. Certain liquid crystal polymers or leuco dyes have phase transitions at specific temperatures (for example, turning from colored to colorless at ~38 °C). A dressing can incorporate such a strip to give a visual cue if the wound becomes abnormally warm (feverish) or cold. However, more precise approaches use electronic temperature sensors. Tiny thermistors or Resistance Temperature Detectors (RTDs) can be embedded in a dressing to provide continuous temperature readings [35]. Additionally, infrared (IR) thermography stickers (which absorb IR from the skin and display a temperature reading) have been trialed for wounds—though getting accurate readings through a dressing can be tricky unless the dressing is IR-transparent or has a window [36].

**Figure 2:**
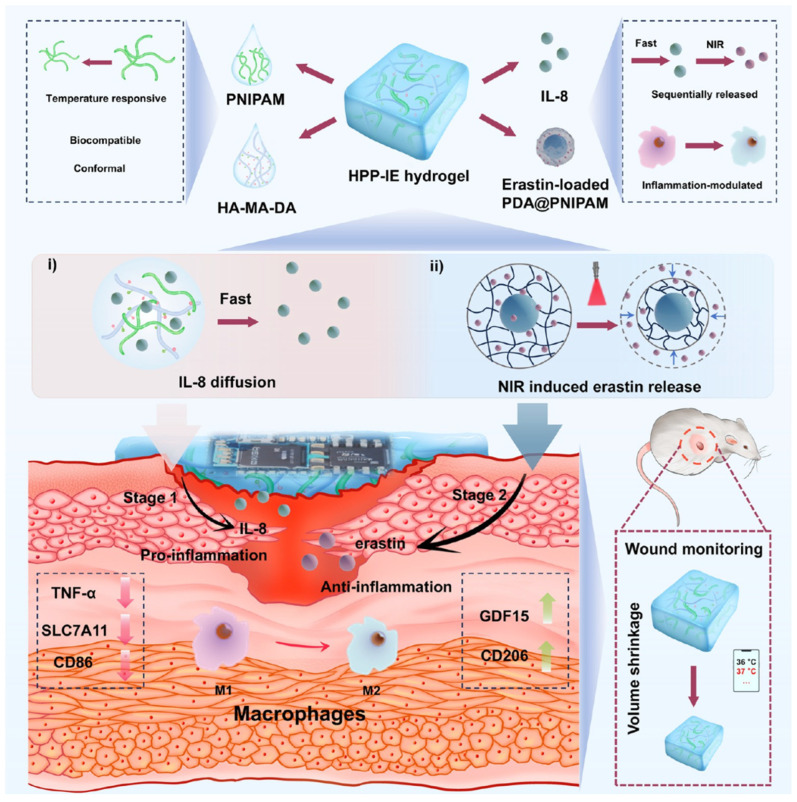
(**i**) First stage of sequential drug release: rapid diffusion and release of IL-8 from the composite hydrogel matrix, (**ii**) second stage of sequential drug release: NIR-driven release of erastin from PDA@PNIPAM nanoparticles. Reprinted from Ref. [38].

For responsive actuation, thermo-responsive polymers play a key role. The most commonly studied is Poly(N-isopropylacrylamide) (PNIPAm), a hydrogel-forming polymer with a Lower Critical Solution Temperature (LCST) around 32–34 °C (close to skin temperature). Below the LCST, PNIPAm hydrogels are swollen with water; above it, they expel water and collapse (a volume phase transition) [37]. By tuning PNIPAm or using copolymers, one can set the transition to around 37–40 °C, so that the polymer dramatically changes properties if the wound area gets a mild fever. How can this help a wound? One example is two-stage drug release: A PNIPAm-based dressing can be engineered to hold back a certain drug at normal temperature but release it rapidly when the polymer collapses at higher temperatures. Qiu et al. designed a dual-layer hydrogel where the inner layer had PNIPAm loaded with antibiotics, and the outer layer was a temperature sensor; when the wound’s temperature rose by >2 °C, the PNIPAm layer shrank and expelled a burst of antibiotic into the wound. Concurrently, the integrated sensor signaled the event [38]. This kind of feedback loop ensures drug is delivered right when infection is heating up the wound (Figure 2).

Materials for thermal sensing can also include phase-change nanoparticles. For instance, nanoparticles that melt at a certain temperature will change the optical properties of the dressing (like turning a transparent film opaque). Researchers have embedded such particles (e.g., fatty acid particles that melt at 38 °C) in dressings to make a simple temperature threshold indicator: below 38 °C they scatter light (dressing looks opaque white), above 38 °C they melt [39], and the dressing turns clear, signaling high temperature. This is somewhat experimental but could allow at-a-glance checks for wound “fever.”

In conclusion, temperature-responsive intelligent dressings serve as both thermometers and thermostats for chronic wounds. They ensure the wound stays in an optimal thermal range and use temperature as a trigger for therapeutic actions. By correlating with infection and healing status, temperature data from smart dressings can guide timely decisions [40]. As with pH, temperature is often part of a bigger sensing suite; the synergy of multi-parametric sensing (pH + temp + others) can greatly improve reliability (for instance, infection usually causes both pH rise and temperature rise, so detecting both increases confidence). The reason we highlight temperature-responsive materials is that temperature is already a vital sign in wound assessment—making this approach intuitively accepted by clinicians. Moreover, polymers like PNIPAm are well-studied and relatively easy to incorporate into hydrogels, suggesting these systems can be scaled up. Some recent prototypes have advanced to animal testing with positive results, indicating translational potential. Future research may see combined pH-temperature responsive dressings, e.g., hydrogels that only release drug when both conditions of high pH and high temperature are met, further refining targeted therapy.

### 3.3. Moisture-Responsive Dressings

Maintaining proper moisture is a cornerstone of wound healing—the dictum “moist wound healing” stems from Winter’s finding that occluded (moist) wounds re-epithelialize faster [41]. However, balance is key: too much exudate macerates surrounding skin and can harbor bacteria, whereas too little moisture desiccates the wound bed and impedes cell migration [42]. Chronic wounds often have heavy exudate due to prolonged inflammation or, conversely, may have necrotic dry tissue. Moisture-responsive dressings aim to sense and manage the wound’s hydration levels in real time [43].

Conventional advanced dressings like hydrocolloids and foams already manage moisture by absorbing exudate while keeping the wound moist. Intelligent moisture-responsive designs add a layer of sensing, indicating when the dressing is saturated or the wound is too wet/dry [44]. One approach is colorimetric humidity indicators. Some dressings incorporate a small window with a humidity-sensitive dye that changes color when relative humidity under the dressing goes beyond a threshold, signaling that exudate level is high [45].

A more technological approach uses impedance or capacitance sensors. Wound exudate is conductive (rich in ions and proteins), so as a dressing soaks it up, its electrical impedance changes. Researchers have developed thin conductive threads or printed electrodes in dressings that act like moisture sensors: as they get wetter, their impedance drops. By measuring this, one can quantify how saturated the dressing is [46]. For instance, a smart dressing might have two conductive tracks printed on a transparent film; when dry, there’s almost no current between them, but as saline or exudate bridges them, current flows indicating moisture presence. One commercially launched device, WoundSense, uses an impedance-based sensor pad that is placed under a regular dressing; a handheld reader can be connected to measure dressing saturation without removing it [47]. It provides a numerical moisture level, helping clinicians decide if the dressing needs changing.

Flexible capacitive sensors are another tool. A parallel-plate capacitor’s capacitance increases with the dielectric constant of the medium between plates [48]. Dry dressings (air) have a certain capacitance; when filled with fluid (higher dielectric constant than air), capacitance goes up. Flexible capacitive patches have been integrated into foam dressings so that a quick scan (via RFID or similar) can tell how much fluid has been absorbed, based on capacitance changes [49].

In summary, moisture-responsive smart dressings ensure the wound is in that “Goldilocks” zone of moisture. They extend the concept of moisture control by making it dynamic and feedback-driven. For chronic wounds, this directly addresses problems of maceration (which can cause wound expansion and fungal infections of surrounding skin) and desiccation (which stalls healing). Combined with other smart features, a moisture-responsive dressing contributes to a more autonomous wound care system where the dressing itself can say “I need changing” or even adapt to keep conditions ideal. The reason we highlight temperature-responsive materials is that temperature is already a vital sign in wound assessment—making this approach intuitively accepted by clinicians. Moreover, polymers like PNIPAm are well-studied and relatively easy to incorporate into hydrogels, suggesting these systems can be scaled up. Some recent prototypes have advanced to animal testing with positive results, indicating translational potential.

### 3.4. Pressure-Sensing Dressings

Chronic wounds like pressure ulcers (bedsores) and diabetic foot ulcers are often precipitated or exacerbated by sustained pressure, friction, or shear that compromises local blood flow [50]. Offloading pressure is critical for healing—for example, offloading a diabetic foot ulcer with special boots or cushions, or regularly turning a bedridden patient to prevent pressure ulcer formation. Pressure-sensing dressings come into play as tools to monitor and potentially alleviate harmful pressure at the wound interface [51].

These smart dressings embed flexible pressure sensors that can map the pressure distribution on the wound or at-risk area. Several sensor technologies are used: Piezoresistive sensors, Capacitive sensors, Piezoelectric sensors and Triboelectric sensors [52].

Currently, commercial availability of pressure-sensing dressings is limited. Some start-ups and research consortia have prototypes, but none is widely on the market as a packaged dressing. The concept may gain traction as part of “smart patient monitoring” in hospitals, and once proven, it could integrate with dressings [53].

In summary, pressure-sensing smart dressings extend wound care into the biomechanical realm, ensuring that chronic wounds (and at-risk areas) are not subjected to the mechanical stresses that impede healing. They act as continuous watchdogs against a key extrinsic factor in wound chronicity. By alerting healthcare providers or patients to offload pressure in a timely manner, they can significantly contribute to better healing outcomes and prevention of further tissue damage. As sensor technology continues to advance and wireless systems become cheaper, it’s likely we’ll see pressure-sensing capabilities bundled into comprehensive smart wound care systems for bed-bound and diabetic patients in the near future.

Although still emerging, pressure-sensing dressings are included because mechanical stress is a major cause of chronic wounds (and their recurrence). Sensing and mitigating pressure is thus practically important, especially for immobile or insensate patients. The technology to do so (flexible pressure sensors) exists and has seen use in other wearables. Translationally, adding a thin pressure sensor layer to a dressing or bandage is feasible. The barrier is more in demonstrating that alerts lead to improved outcomes, but given the known benefit of pressure relief, this approach is likely to be valuable. As healthcare moves toward continuous monitoring, pressure-sensing wound dressings fit well into that paradigm and have clear clinical relevance in preventing and treating pressure-related wounds.

### 3.5. Sustained and On-Demand Drug-Releasing Dressings

**Figure 3:**
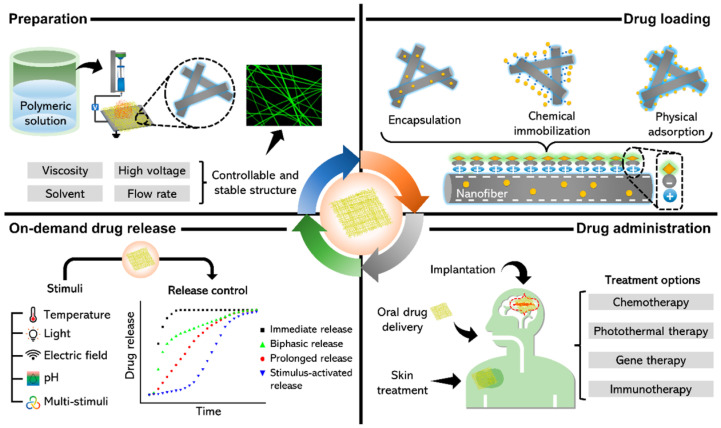
Schematic of on-demand drug delivery systems using stimuli-responsive NFs. Reprinted from Ref. [56].

Many chronic wounds fail to heal due to high bioburden (infection), chronic inflammation, or lack of growth factors. Delivering therapeutic agents directly to the wound can improve healing—for instance, antimicrobials to control infection, or cytokines to modulate inflammation [54]. Traditional dressings can be impregnated with drugs (like silver or iodine dressings, or alginate with antibiotics), but they release drugs in a mostly uncontrolled fashion (burst release followed by gradual decline) and cannot adjust to the wound’s changing needs. Intelligent drug-releasing dressings aim to provide controlled, sustained, or even feedback-triggered release of therapeutics to the wound over time [55]. For example, Rezk et al. report a bilayer electrospun membrane—outer PCL layer and inner PDO loaded with TiO_2_ nanoparticles and tetracycline [56], that delivers an initial burst followed by ~4-day controlled release and shows strong antibacterial activity against *E. coli* and *S. aureus*, highlighting feasibility for antibacterial dressings (Figure 3).

Hydrogels have been heavily researched as controlled-release wound dressings. Their three-dimensional polymer networks can entrap drugs and release them by diffusion or network degradation. By modifying crosslink density or using polymers that degrade under specific conditions, one can tailor the release profile. Sustained-release hydrogels slowly elute drugs over days, maintaining therapeutic levels in the wound [57]. Such dressings reduce the frequency of drug application compared to daily antibiotic ointments, thus simplifying care and ensuring consistent drug presence.

However, the cutting edge is stimuli-responsive release, where the dressing releases its payload on demand in response to environmental triggers (some of which we covered like pH or temperature). Apart from those triggers, other interesting stimuli include enzymes, light, electric fields, and magnetic fields. We saw an example with enzymes (β-lactamase-triggered release) [56]. Additionally, iron oxide nanoparticles in a dressing can heat up under an external magnetic field (magnetothermal effect), which can then cause a phase change or increased diffusion to release drugs. Researchers used this to create a remotely triggerable dressing: a hydrogel with antibiotic and Fe_3_O_4_ nanoparticles (Figure 3). When a magnetic field was applied outside the bandage, the particles heated slightly and the hydrogel’s mesh loosened, resulting in a burst of antibiotic release [58].

Also, intelligent drug-releasing dressings must balance keeping the drug stable vs. releasing it when needed. Many biological agents (growth factors, peptides) are unstable in a wound’s protease-rich environment. Encapsulation techniques (like nanoparticles, liposomes) are used to protect them until release [16]. Also, loading enough drug into a thin dressing and releasing it over long durations is challenging; some approaches include layer-by-layer coatings that can carry high drug loads and peel away gradually.

In summary, intelligent drug-releasing dressings represent a therapeutic leap—turning the passive covering into an active drug delivery system that adapts to the wound’s needs. For chronic wounds, which often require long-term topical therapies, these systems could maintain optimal therapeutic levels continuously, reduce systemic side effects, and dynamically address infection and tissue regeneration phases. When combined with sensors (e.g., releasing drugs only when sensors indicate infection or high inflammation), they embody the essence of closed-loop medicine at the wound site. As these technologies progress, we anticipate treatments that, for example, sense a spike in protease levels in a chronic wound and release a protease inhibitor, or sense colonization by pathogenic bacteria (perhaps via detecting their unique enzymes or toxins) and respond with a timely dose of antimicrobial—thereby staying one step ahead of wound deterioration.

### 3.6. Electroactive Dressings

Electricity has long been known to influence wound healing—in fact, normal skin injury generates an endogenous electric field (the “skin battery”) that guides cell migration (a phenomenon called galvanotaxis) [59]. In chronic wounds, this natural electric signaling is often diminished or disoriented, contributing to stalled healing. Additionally, electrical stimulation can combat infection and modulate inflammation. Electroactive dressings leverage these principles by delivering electrical stimuli or facilitating electrochemical interactions at the wound site [60]. They include conductive dressings (which act as electrodes or distribute current) and electrostimulative dressings (which may generate electric fields or currents themselves) [61].

Electrical stimulation (ES) of wounds typically uses low-intensity currents (microampere to milliampere range) or electric fields applied periodically [3]. Mechanistically, ES is known to: (1) attract repair cells (keratinocytes, fibroblasts) to the wound edge (since these cells migrate towards the cathode in an electric field) [62]; (2) increase local blood flow (vasodilation) which improves oxygen and nutrient delivery [63]; (3) enhance collagen synthesis and promote a more organized extracellular matrix; and (4) disrupt bacterial biofilms and kill microbes (electric fields can make bacteria more permeable). The result is often accelerated wound closure, improved tissue quality, and reduced infection [64].

Traditional ES therapy for wounds involved placing electrode pads around the wound and using an external device (e.g., pulsed electromagnetic field or TENS unit). Electroactive dressings integrate this capability into the dressing itself [65].

Conductive hydrogel dressings (CHs) are a key innovation [66]. These are hydrogels enriched with conductive elements—such as carbon nanotubes, graphene, metal nanoparticles (like silver nanowires), or intrinsically conductive polymers (polypyrrole, PEDOT:PSS) [67]. The hydrogel provides a moist wound environment and biocompatibility, while the conductive additives allow electrical signals to pass uniformly across the wound [68]. CHs can serve as a “smart electrode” conforming to the wound. Instead of a rigid electrode, the entire gel is conductive, ensuring even distribution of current and intimate contact with the tissue [3]. This avoids hotspots of high current that could cause burns and mitigates the hazard of high voltages because the resistance is low and spread out [69]. CHs can be connected to external circuitry for programmed stimulation, or they can incorporate self-powered elements [68].

Self-powering can be achieved through piezoelectric components that generate small currents when flexed by body movements, or bio-batteries that harness ionic differences in wounds [70]. One fascinating design printed arrays of silver and zinc microparticles on a polymer that, when moistened by wound fluid, creates micro-batteries producing ~0.5–1 V (the principle behind Procellera). These microcurrents (in the microamp range) mimic the skin’s natural electric fields and were shown to enhance keratinocyte migration and closure in studies [71]. Essentially, the dressing generates a continuous electric field as long as it’s moist—a perfect synergy since wounds are moist environments [72]. Another strategy is triboelectric nanogenerators: some experimental bandages have layers of different materials that, with motion (like patient breathing or moving), produce electrical pulses that can stimulate the wound [73].

**Figure 4:**
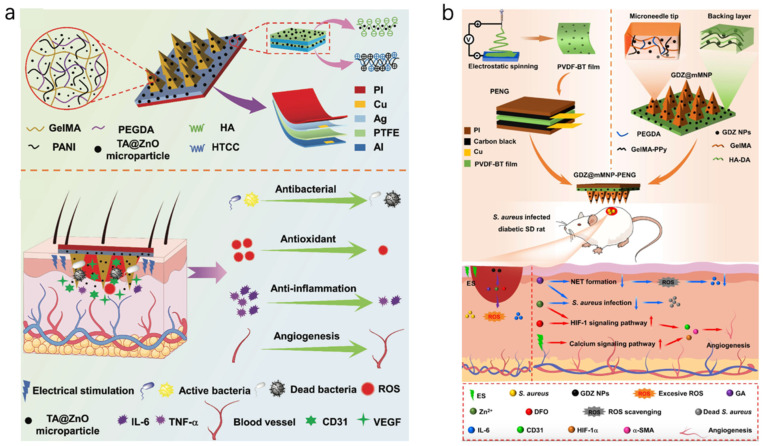
(**a**) device schematic of TZ@mMN-TENG. Reprinted with permission from Ref. [74] (**b**) The schematic illustration for the design and preparation of the GDZ@mMNP-PENG for infected diabetic wound healing. Reprinted with permission from Ref. [75].

Numerous studies document how conductive dressings with ES promote chronic wound healing. Recently, Li et al. introduced an integrated self-powered microneedle platform (TZ@mMN-TENG) specifically engineered for the treatment of infected diabetic wounds (Figure 4a). The system employed a microneedle array loaded with tannin@ZnO microparticles, endowing the dressing with potent antibacterial, antioxidant, and anti-inflammatory properties. Simultaneously, a built-in triboelectric nanogenerator (TENG) harvested biomechanical energy to generate exogenous electrical stimulation in a fully self-sustained manner. The incorporation of polyaniline (PANI)-doped conductive microneedles enabled not only precise therapeutic delivery but also efficient electrical conduction. In a full-thickness diabetic rat wound model infected with Staphylococcus aureus, the TZ@mMN-TENG markedly accelerated tissue repair, achieving over 99% bacterial eradication, enhanced collagen deposition, and significant downregulation of pro-inflammatory cytokines (TNF-α, IL-6). Moreover, it facilitated robust angiogenesis by upregulating CD31 and VEGF expression, demonstrating a synergistic combination of antimicrobial, anti-inflammatory, and pro-regenerative effects [74]. Li et al. also constructed a piezoelectric nanogenerator (PENG) based on PVDF-barium titanate (PVDF-BT) nanofiber membranes, allowing efficient energy harvesting from subtle mechanical movements. The microneedles delivered a synergistic combination of gallic acid, deferoxamine (DFO), and ZIF-8 nanoparticles (GDZ NPs), offering potent antibacterial, antioxidative, anti-inflammatory, and pro-angiogenic activities. Proteomics analysis revealed that the GDZ@mMNP-PENG not only promoted HIF-1-mediated angiogenesis but also remodeled the inflammatory microenvironment by downregulating neutrophil extracellular trap formation (Figure 4b). Those platforms combine antimicrobial therapy, redox modulation, and bioelectric stimulation in a single, compact microneedle system that is fully self-powered by endogenous mechanical movements, avoiding the need for external energy inputs and enhancing patient compliance [75].

Another notable advancement in this domain was reported by Cao et al., who fabricated a three-dimensional printed bilayer dressing aimed at improving diabetic wound repair. The construct comprised a basal layer of reactive oxygen species (ROS)-responsive polyurethane nanofibers encapsulating doxycycline, combined with an upper hydrogel layer made from a quaternized polysaccharide-based ionic conductive polymer. This dual-layer configuration enabled site-specific drug release triggered by elevated ROS concentrations, while the conductive hydrogel concurrently facilitated electrical stimulation (ES) and modulated macrophage polarization toward a regenerative M2 phenotype. When evaluated in diabetic rat models, the composite dressing achieved markedly superior outcomes relative to single-layer controls, including accelerated wound closure, greater collagen matrix formation, and enhanced neovascularization [76].

In summary, electroactive smart dressings provide a multifaceted assault on the barriers to chronic wound healing. They restore or amplify the body’s bioelectric signals to direct cell behavior and suppress infection, effectively “jumpstarting” stalled wounds. The integration of conductive biomaterials like CHs ensures that these electrical therapies are delivered safely and uniformly. The evidence to date consistently points to improved healing rates and quality with electrotherapy, and intelligent dressings are making this therapy more accessible and automated.

### 3.7. Biosensor-Integrated Dressings and Digital Health Integration

Perhaps the most encompassing category of intelligent wound dressings are those that integrate biosensors—devices that detect specific biological or physical parameters—into the dressing, often with connectivity for data transmission [77]. These “smart bandages” aim not only to treat the wound but also to provide continuous, objective monitoring of the wound’s status. In the age of digital health and telemedicine, such technology can enable remote wound management, early warning of complications, and personalized treatment adjustments.

A variety of sensors have been researched for incorporation into dressings, including chemical sensors, physical sensors, optical sensors/imagers and microbial sensors [78]. The key is making these sensors flexible and biocompatible. Many research efforts use printed electronics on polymer films or even directly on fabric/hydrogel. Advances in stretchable circuits allow inclusion of microprocessors and communication chips on a dressing that can bend and stretch with the body.

An intelligent dressing with biosensors essentially creates an “Internet of Things” (IoT) device on the wound. Typically, a small microcontroller in the dressing collects sensor data and then communicates it wirelessly via Bluetooth or NFC to a nearby device (like the patient’s smartphone or a dedicated reader) [3]. From there, data can be uploaded to the cloud for clinicians to review. In hospital settings, a dressing might connect to a central monitoring system.

For example, a next-gen smart dressing described by Gao et al. includes sensors for pH, temperature, uric acid (a marker of wound oxidative stress), and a strain gauge, all connected to a tiny Bluetooth module and battery on the bandage’s edge. Every several minutes, it sends a data packet with current readings. If any parameter goes out of the preset normal range (say pH jumps indicating infection), it can send an instant alert to the patient’s phone and the clinic [21]. This continuous stream of data enables trend analysis. Instead of only seeing a wound during weekly visits, clinicians can observe how pH, temperature, etc., are evolving hour-by-hour, which could inform if a treatment is working or if an intervention is needed sooner. It’s like having vital signs for the wound. Moreover, these data can feed into predictive algorithms (AI) [79]. There is growing interest in using machine learning to predict wound healing outcomes. With continuous multi-sensor data, an AI could potentially detect subtle patterns that precede wound healing or deterioration—for example, a combination of rising temperature and pH plus increasing exudate impedance might predict infection 24 h before full-blown symptoms. Early studies are combining smart bandage data with AI to create a “wound forecast” that can guide personalized care.

**Figure 5:**
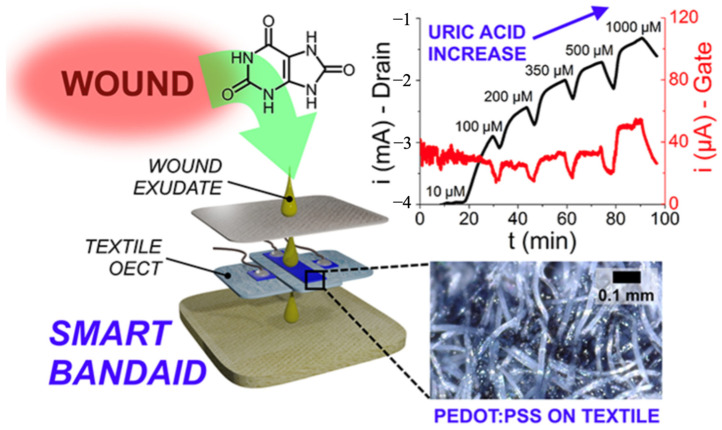
Smart Bandaid Integrated with Fully Textile OECT for Uric Acid Real-Time Monitoring in Wound Exudate. Reprinted with permission from Ref. [80].

Another prototype integrated a fully textile organic electrochemical transistor (PEDOT: PSS-based) into a smart bandage for real-time uric acid monitoring in wound exudate [80]. Using medical-grade textiles and passive sampling, it quantified UA across the clinically relevant range under flow and wirelessly streamed data to a smartphone app (Figure 5). While single-analyte, this label-free, low-voltage platform shows a practical path to connected, disposable dressings.

In conclusion, biosensor-integrated intelligent dressings are the epitome of marrying wound care with modern technology. For chronic wounds, which benefit from careful monitoring and timely intervention, these dressings provide a constant watch and even first-line responses, bridging the gap between clinic visits. As part of the broader trend of digital health, they enable more precise and personalized wound management—a far cry from the one-size-fits-all gauze of the past. With continuing advancements, patients with chronic wounds might soon wear a bandage that not only helps heal their wound but also essentially reports on its condition and progress 24/7, guiding both patient and clinician in making optimal care decisions.

This category is included because it represents the future vision of wound care—where dressings are not just passive or even just reactive, but fully interactive and connected. The practical relevance is enormous: chronic wounds often require frequent check-ups; a biosensor dressing could reduce clinic visits by safely extending monitoring into the home. The data-driven approach aligns with modern healthcare trends (remote patient monitoring, personalized medicine). Technologically, while complex, it leverages the ongoing advances in flexible electronics and IoT. Scalability will depend on cost reduction of sensor components, but given the ubiquity of smartphones and Bluetooth, much of the infrastructure already exists. The challenge is to ensure reliability and user-friendliness, but early results are promising, and some simpler versions (like colorimetric sensor apps) might reach practice soon. Overall, biosensor-integrated dressings underscore the paradigm shift from treating wounds reactively to managing them proactively with continuous insight.

### 3.8. Shape-Memory and Self-Adapting Dressings

Shape-memory materials (SMMs) can “remember” a predetermined shape and revert to it when triggered by a stimulus (often heat). In wound care, shape-memory dressings exploit this property to achieve mechanical actions like wound closure, compression, or conformal filling of wound cavities. Chronic wounds, especially large or irregular ones, can benefit from such adaptive mechanical support—something static dressings cannot provide [81].

Polyurethane-based SMPs are common because their transition temperature can be tuned around 37 °C by adjusting hard/soft segment chemistry [82]. A study developed SMP polyurethane strips as wound closure devices—these were stretched at low temperature, placed across an incisional wound like sutures, and as they warmed, they contracted, bringing the wound edges in apposition [83]. Essentially, they functioned like smart adhesive sutures. In pig skin incisions, they achieved closure without the need for staples, and because the force was distributed along the strip, it minimized tissue damage and resulted in fine scars.

**Figure 6:**
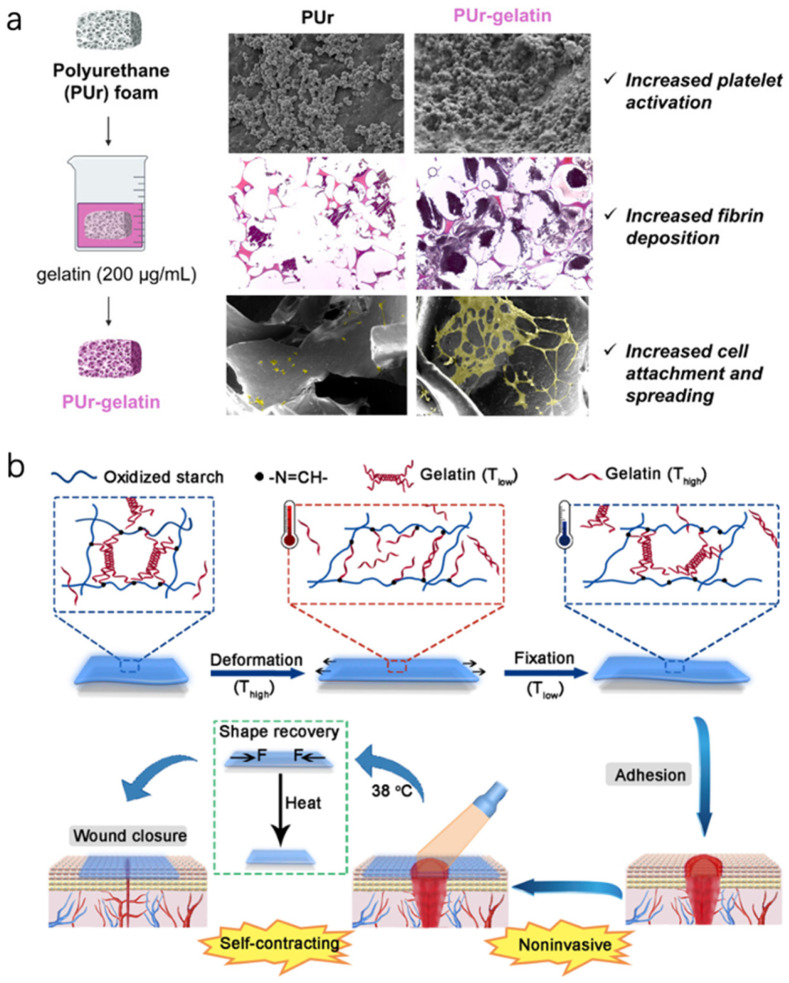
(**a**) Bioactive Polyurethane Shape Memory Polymer Foam Dressings with Enhanced Blood and Cell Interactions for Improved Wound Healing. Reprinted from Ref. [85] (**b**) Schematic representation of self-contracting OSG for noninvasive wound closure and healing. Reprinted from Ref. [84].

A scenario for chronic wounds is an SMP foam that is compressed (like a small sponge) for insertion into a deep wound cavity, then body heat causes it to expand and conform to the cavity, filling dead space and applying a gentle pressure that can help stop bleeding or provide support [84]. One study reported a bioactive SMP foam that expands at 37 °C and also releases phenolic compounds [85]; it demonstrated excellent hemostasis in a traumatic wound model and then later could be easily removed after recooling (the foam stiffens when cooled, making it easier to pull out in one piece). For chronic wounds, such foams could combine debridement and filling—inserted cold to scrape out debris as they expand, and then providing a matrix for tissue to grow into (Figure 6a). Another example is an oxidized starch/gelatin hydrogel that was cast in a stretched state, then dried. Upon rehydration at body temperature, it “remembers” a contracted state and shrinks in area [84]. Placed on a wound, it could thereby exert a contractile force. Researchers showed this self-contracting hydrogel reduced wound area significantly more than a non-shrinking hydrogel in rat wounds, effectively acting like a synthetic wound contracture aiding closure (Figure 6b).

In conclusion, shape-memory intelligent dressings bring a novel element of mechanical intelligence to wound care—they physically adapt and change shape to aid healing. For chronic wounds, especially large ones, they can reduce the burden on the body by mechanically converging the wound, potentially reducing healing time and scar size. They can also reach places and conform in ways that static materials cannot, improving contact and therapeutic delivery. While currently mostly in experimental or early translational stages, the substantial benefits observed in preclinical studies (significantly faster wound closure, etc.) suggest that with further development, these could become valuable adjuncts for chronic wound management.

## 4. Current Limitations and Challenges

Despite the exciting potential and encouraging preclinical results of intelligent wound dressings, several challenges must be addressed before they become standard in clinical practice. These limitations span technical, biological, regulatory, and practical domains:

Biocompatibility and Safety

Smart dressings introduce novel materials (nanoparticles, conductive polymers, electronic components) into direct contact with wound tissue. Ensuring that these do not provoke adverse reactions is paramount [86]. Toxicity of some nanomaterials (e.g., carbon nanotubes, certain quantum dots, high levels of metal ions) is a concern; chronic wounds have prolonged exposure, so even low-level cytotoxicity can accumulate. For instance, while silver is antimicrobial, too much silver release can impair cell growth—thus sustained silver-releasing dressings must modulate dosing carefully. Conductive polymers may contain residual monomers or dopants that could be inflammatory [87]. Rigorous biocompatibility testing is needed for each new component.

An important aspect is the fate of materials over time. Many smart dressings are designed to be biodegradable or at least not permanent. Degradation products must be non-toxic. For example, polylactic acid-based fibers will break down to lactic acid, which in excess could lower local pH and cause irritation if not buffered [88]. Ensuring degradation is slow and metabolites are safely absorbed or neutralized is key. Similarly, repeated application of nanoparticle-laden dressings raises questions about nanoparticles accumulating in tissue or being absorbed systemically. So far, studies indicate materials like graphene (when properly functionalized) and polymers like PEDOT: PSS are reasonably biocompatible in wound contexts, and gold nanoparticles are generally inert, but others like Ag or Cu can exhibit dose-dependent cytotoxicity. Long-term safety data in humans are limited at this stage, given that most smart dressings have only been tested in labs or small animal studies.

Electrical stimulation dressings must be safe regarding current levels—excessive current can cause burns or nerve/muscle stimulation. Intelligent systems need fail-safes so that if a sensor error occurs, the device doesn’t, say, keep ramping up current [89]. The Stanford bandage, for example, used very low currents and they caution about scaling up size (the human-scale device must spread current appropriately to avoid hotspots).

For biosensor dressings, accuracy and reliability are part of safety: a false negative (failing to detect an infection) could lead to missed treatment, or a false positive (triggering unnecessary drug release or alarm) could cause overtreatment or alarm fatigue. Ensuring sensor calibration and stability in the wound’s harsh environment (fluctuating enzymes, proteases, pH, and biofouling by proteins or bacteria) is challenging. Biofouling in particular can drift sensor readings over time—e.g., a glucose sensor might get coated by fibrous exudate and under-report levels. Strategies like anti-fouling coatings or periodic calibration routines (maybe auto-zeroing the sensor) are being explored [90].

In summary, safety and biocompatibility considerations require that smart dressing developers not only optimize function but also thoroughly assess any adverse effects. Many studies now include cytotoxicity assays, skin irritation tests, and analysis of inflammatory markers to ensure a new dressing doesn’t trade one problem for another. Regulatory bodies will scrutinize these aspects; thus, demonstrating that materials either stay largely local without systemic harm, or have an established safety profile (like components already used in other implants), will smooth the path to approval. For example, using FDA-approved polymers (PLGA, chitosan, etc.) as the base materials can alleviate safety concerns, as their biodegradation and biocompatibility are known.

Complexity and Reliability

With increased functionality comes increased complexity. Intelligent dressings are essentially medical devices rather than simple wound covers. They have multiple points of potential failure: battery life can end, circuits can break (especially on a moving body part), sensors can degrade, drug reservoirs can deplete or leak, shape-memory components might not actuate correctly every time, etc. For clinicians to trust and adopt these, they must be highly reliable and user-friendly. A nurse should be able to apply one without needing an engineering manual—thus, a lot of effort is being made in design is toward simplification and integration [91].

Many prototypes are assembled by hand in labs; translating to manufacturing is non-trivial. Printing flexible electronics and embedding them consistently will require advanced fabrication techniques. Ensuring the dressing still functions after being bent, slept on, exposed to wound fluid, etc., is a significant engineering challenge. Water-proofing electronics while allowing moisture transmission from the wound (for breathability) is a delicate balance.

Cost and Accessibility

High-tech dressings will inevitably cost more than standard ones, at least initially. Chronic wound care already is expensive, and convincing payers to reimburse these advanced products will depend on proving they significantly improve outcomes or reduce overall costs (e.g., by shortening healing time, preventing hospitalizations for infection, or reducing clinic visits). Economic analyses will be needed. The materials themselves (nanoparticles, electronics) can be pricey, although mass production and Moore’s law might lower some costs.

For widespread use, ease of use is critical. Many chronic wounds are cared for at home by patients or family. If a smart bandage requires complex pairing with an app or careful sensor calibration daily, it might not be feasible for all patients (consider elderly patients with limited tech familiarity). Designers are thus trying to make them as “plug-and-play” as possible: e.g., colorimetric outputs or automatic data uploads that the patient doesn’t have to fiddle with.

Regulatory and Clinical Validation

Intelligent dressings often straddle the line between devices and drugs (as combination products). Regulatory approval processes can be more involved: they may need demonstration of safety and efficacy through robust clinical trials, which are time-consuming and costly. Regulatory bodies like the FDA will consider issues such as electrical safety, software reliability (for any algorithms used), and risk of misuse. While the FDA has pathways for combination wound products, gathering the required preclinical and clinical data is a hurdle. Historically, some seemingly promising wound treatments (like certain growth factor therapies) faltered in large trials because the real-world benefit was modest. So, proving that adding all this intelligence truly makes a significant difference (e.g., healing 2x faster, or X% more wounds healed) is essential to justify regulatory approval and clinician adoption.

Additionally, there are standardization issues: Wound assessment is partly subjective (like how to measure wound improvement). Smart dressings can provide objective data, but correlating that to meaningful clinical endpoints (e.g., complete healing or wound size reduction) requires careful study design in trials. There’s also the question of training: clinicians might need training to interpret new kinds of data (like what does a certain pH pattern mean clinically, or how to respond to a smart bandage alert appropriately).

Patient Factors

Chronic wound patients often have comorbidities (diabetes, neuropathy, vascular disease) and may not notice or feel subtle differences. If a device malfunctions or causes mild discomfort, they might not be aware due to neuropathy, which underscores the need for devices to fail safe (e.g., if a sensor fails, it should alert or stop active therapy).

Furthermore, not all patients may accept a more complex dressing—some might be anxious about having “electronics” on them or doubt its benefit. Proper patient education and demonstration of benefits will be needed to improve acceptance.

Equitable Access

It’s important that these advanced therapies not widen the disparity gap in healthcare. Many chronic wound sufferers are older or in lower socioeconomic groups (like elderly diabetics or immobile patients). If smart dressings remain very expensive or only available in cutting-edge centers, then many who need them won’t get them. Efforts to drive down costs and demonstrate cost-effectiveness to insurance (so they will cover them) are needed to ensure broad access.

In summary, the road to mainstreaming intelligent wound dressings involves overcoming these challenges by iterative design improvements and rigorous testing. We are in the early phases of translation: a lot of the focus now, beyond proving concepts, is making these technologies robust, user-friendly, and clinically validated. Encouragingly, multidisciplinary collaborations (engineers, biologists, clinicians, regulatory experts) are actively tackling these issues. As these hurdles are cleared, we can expect more and more pilot programs in hospitals using smart dressings, leading eventually to routine care for chronic wounds with a new generation of “smart” products [92].

## 5. Future Directions in Intelligent Wound Dressings

The field of smart wound dressings is evolving rapidly, and several exciting directions are on the horizon. Many current prototypes excel in one domain (sensing or drug delivery or stimulation). The next generation will likely combine multiple intelligent functions in one dressing. For example, “all-in-one” smart bandages that monitor key wound parameters (pH, temp, infection markers), deliver drugs as needed, and provide electrical/mechanical stimulation are a logical progression. Such integration will require careful coordination (ensuring one function doesn’t interfere with another) and advanced fabrication (layering sensors, circuits, and drug reservoirs in a thin format). Research is already moving that way—e.g., a recent prototype integrated sensor, controlled drug release, and electrical stimulators in one patch for diabetic ulcers [36]. These holistic devices could essentially act as an “artificial wound healing assistant,” handling multiple aspects of care autonomously [93].

Also, with the influx of data from biosensor dressings, AI will play a key role in interpreting it. Future smart dressings might incorporate onboard simple AI (for instance, an algorithm that learns a patient’s baseline wound readings and detects anomalies), or more likely, they’ll feed data to cloud-based AI that can compare against large datasets of wound healing trajectories. AI could predict, for example, if a wound is likely to heal with current treatment or if it might deteriorate, enabling proactive adjustments. Additionally, AI could optimize treatment regimens: e.g., learning from sensor data when is the best time to deliver a drug dose or apply electrical stimulation for maximal effect. The integration of AI was highlighted in recent reviews as a frontier, sometimes termed “smart wound care 2.0”—using machine learning on wound images and sensor streams to guide personalized therapy [94].

Researchers will also likely devise sensors for biomarkers not yet tapped. For instance, monitoring inflammatory cytokines (like IL-6, TNF-α) or protease activity in real time could directly measure the chronic inflammatory state of a wound. There’s already progress on small aptamer-based sensors for such proteins that could be put on a bandage. Also, genetic sensors might detect RNA or DNA from infecting bacteria (for rapid identification of pathogen type or antibiotic resistance genes in the wound). Another interesting area is mechanobiological sensing—e.g., sensors that detect wound tissue stiffness (which changes as scar tissue forms). This could inform whether the wound is healing with too much fibrosis (and maybe trigger an anti-fibrotic drug release).

Beyond current stimuli (pH, temp, etc.), future smart dressings may respond to novel triggers like specific enzymes or even biomagnetic signals from tissue. For therapy, light-based therapies might get integrated: imagine a bandage that has LED arrays to deliver low-level light therapy (LLLT) or photodynamic therapy in response to sensor readings (e.g., if bacterial load is high, turn on a bactericidal light with a photosensitizer). Already, work on LED-incorporated dressings for phototherapy is underway. Similarly, ultrasound is used in wound care (e.g., low-frequency ultrasound debridement)—perhaps a bandage could incorporate piezoelectric elements that deliver ultrasound pulses to break up biofilms or stimulate cells [95].

Additionally, every chronic wound is a bit different (patient comorbidities, cause, microenvironment). Future devices might adapt their behavior to the individual. For example, a smart bandage might “learn” how a patient’s wound responds—if it notices every time, it applies an antibiotic when the pH drops for 2 days and then rises again, it might schedule itself to dose slightly before the usual rise to preempt infection [96]. Or it might adjust electrical stimulation parameters based on how quickly impedance is changing (like a self-optimizing regimen per patient).

Many current smart dressing components are not biodegradable (electronics, some polymers), requiring removal. A future trend could be biodegradable electronics (transient electronics) that can dissolve harmlessly—research in bioelectronics is developing such devices (e.g., magnesium or silicon-based circuits that degrade). For wound care, a fully biodegradable smart system would be ideal: one could leave it on and it would eventually absorb, eliminating painful removal. Likewise, more bioactive materials (like scaffolds that not only sense but also actively support tissue regeneration by providing cell adhesion sites or releasing regenerative signals) will be integrated. For instance, nanofiber meshes that are electrically conductive and also seeded with stem cells or growth factors, providing a physical scaffold plus smart functions—bridging tissue engineering and smart dressing domains.

On the healthcare delivery side, future wound clinics may have dashboards where nurses monitor multiple patients’ smart bandage data on screens (like ICU vital signs) either in hospital or remotely for home patients. Integration with telemedicine could allow specialists to consult on a wound by reviewing its sensor data and images remotely, which is crucial for patients in rural areas or with mobility issues.

As these devices emerge, parallel efforts in educating clinicians on interpreting and using the data will be important. We might see wound care certification programs include training on smart dressing management and data analytics, ensuring that the human element keeps pace with the technology.

In essence, the future of intelligent wound dressings lies in convergence—convergence of multiple technologies (biosensors, drug delivery, biopolymers, microelectronics, AI) into cohesive products, and convergence of those products with the healthcare system’s way of managing wounds. The ultimate vision is a paradigm where chronic wounds are continuously cared for by a “smart assistant” at the wound site, drastically reducing complications and time to heal. Instead of reactive care, it will be proactive and precision-guided. Achieving this will require interdisciplinary innovation and solving present challenges, but the trajectory is clearly set by the advances we are already witnessing.

## 6. Conclusions

Chronic wounds remain a formidable medical and socioeconomic challenge, but the advent of intelligent wound dressings is poised to transform their management. Over the past decade, research has progressed from passive moisture-retaining dressings to dynamic systems that can sense, react, and adapt to the wound’s needs in real time. We have reviewed the major categories of these smart dressings—from pH-sensitive hydrogels that signal infection by a simple color change, to temperature-responsive polymers that release drugs on cue, to electroconductive scaffolds that deliver healing microcurrents, biosensor-laden bandages that transmit wound vital signs, and shape-memory materials that physically aid wound closure. Each of these innovations addresses specific impediments of chronic wounds: detecting subtle signs of infection or ischemia early, providing controlled on-demand therapy, restoring the bioelectric environment, offloading pressure, and mechanically accelerating closure.

Importantly, numerous in vitro and in vivo studies corroborate the benefits of intelligent dressings. They have demonstrated faster healing rates, reduced infection burden, and improved tissue regeneration in various chronic wound models. Early clinical experiences, though limited, are encouraging—indicating that these technologies can be translated to patient care with positive outcomes if implemented thoughtfully.

Yet, as we stand at this exciting frontier, further work is needed to overcome current limitations. We must ensure these sophisticated dressings are robust, safe, and user-friendly, with proven clinical and economic advantages. Regulatory pathways must be navigated with rigorous evidence. Interdisciplinary collaboration will be the linchpin—marrying engineering ingenuity with clinical wisdom and patient-centric design.

The historical trajectory of wound care—from ancient poultices to modern bioengineered skins—teaches us that innovations which truly improve healing and quality of life will ultimately be adopted. Intelligent dressings represent the next leap in that trajectory: moving from passive treatment to active, responsive care. In the near future, it is conceivable that a chronic wound patient’s journey will be vastly improved—instead of frequent clinic visits and empirical treatments, they may simply wear a smart dressing that continuously optimizes their wound environment, alerts them and their clinicians to issues, and delivers personalized therapy around the clock. This would not only speed healing, but also reduce pain, inconvenience, and healthcare costs, thus significantly enhancing patient outcomes and well-being.

In conclusion, intelligent wound dressings embody the convergence of material science, bioengineering, and digital health in service of an age-old medical challenge. They hold the promise of making chronic wounds heal faster, safer, and smarter. As research and development continue to address current challenges and refine these technologies, we anticipate that smart dressings will transition from experimental novelties to indispensable tools in the clinician’s armamentarium, heralding a new era in wound healing—one where the dressing is not just a passive covering, but an active participant in the healing process.

## Figures and Tables

**Table 1:** Major Categories of Intelligent Wound Dressings—Stimuli and Materials.

Dressing Category	Stimulus/Target	Responsive Mechanism	Clinical Stage
pH-Sensitive Dressings	Wound pH changes (infection elevates pH to ~7–9).	Sensing: pH-responsive dyes or polymers change color or properties; Response: indicate infection or trigger drug release in alkaline pH.	Preclinical (laboratory prototype)
Temperature-Responsive	Local temperature shifts (inflammation/infection; ischemia).	Sensing: Thermochromic or thermoresponsive polymers detect temperature; Response: drug release or physical change at a threshold.	Preclinical (animal study completed; not yet in human trials)
Moisture-Responsive	Wound exudate level (too much exudate vs. too dry).	Sensing: Hygroscopic polymers or impedance sensors detect moisture; Response: color change or signal	Commercial use in some products.
Pressure-Sensing	Pressure/shear on wound (especially for pressure ulcers).	Sensing: Flexible pressure sensors embedded to map pressure; Response: alert if pressure exceeds threshold, preventing tissue damage.	Preclinical (prototype demonstrated in vitro; not yet in use)
Drug-Releasing Dressings	Various wound stimuli trigger therapeutic release. Targets infection, inflammation.	Conductive or electronic dressings deliver electrical currents or electric-field stimulation to the wound bed. This can promote cell migration, increase blood flow, reduce bacterial load, and modulate healing signals	Already have FDA-approved product (static release)
Electroactive Dressings	Bioelectric signals or external command triggers microcurrent stimulation.	Painful, pale, dry wounds, punched-out appearance, weak peripheral pulses	Preclinical (animal)
Biosensor-Integrated Dressings	Multiple biomarkers or parameters: e.g., pH, temperature, oxygen	Incorporates miniature biosensors into the dressing to continuously monitor wound status. Data is transmitted (often wirelessly) to caregivers or smartphones.	Early clinical adoption in parts (sensors external to the dressing)
Shape-Memory Dressings	External triggers or programmed shape recovery over time.	Made of shape-memory materials (polymers or alloys) that can be deformed and will return to a predefined shape when activated.	Preclinical (animal)

## Data Availability

The original contributions presented in the study are included in the article, further inquiries can be directed to the corresponding authors.

## References

[1] Gu H., Li H., Wei L., Lu J., Wei Q. (2023). Collagen-based injectable and self-healing hydrogel with multifunction for regenerative repairment of infected wounds. Regen. Biomater..

[2] Derakhshandeh H., Kashaf S.S., Aghabaglou F., Ghanavati I.O., Tamayol A. (2018). Smart Bandages: The Future of Wound Care. Trends Biotechnol..

[3] Fang Y., Han Y., Yang L., Kankala R.K., Wang S., Chen A., Fu C. (2025). Conductive hydrogels: Intelligent dressings for monitoring and healing chronic wounds. Regen. Biomater..

[4] Farahani M., Shafiee A. (2021). Wound healing: From passive to smart dressings. Adv. Healthc. Mater..

[5] Las Heras K., Igartua M., Santos-Vizcaino E., Hernandez R.M. (2020). Chronic wounds: Current status, available strategies and emerging therapeutic solutions. J. Control. Release.

[6] Rezvani Ghomi E., Niazi M., Ramakrishna S. (2023). The evolution of wound dressings: From traditional to smart dressings. Polym. Adv. Technol..

[7] Bülbül E.Ö., Okur M.E., Okur N.Ü., Siafaka P.I. (2022). Traditional and Advanced Wound Dressings: Physical Characterization and Desirable Properties for Wound Healing. Natural Polymers in Wound Healing and Repair.

[8] Pang Q., Yang F., Jiang Z., Wu K., Hou R., Zhu Y. (2023). Smart wound dressing for advanced wound management: Real-time monitoring and on-demand treatment. Mater. Des..

[9] Qiao B., Pang Q., Yuan P., Luo Y., Ma L. (2020). Smart wound dressing for infection monitoring and NIR-triggered antibacterial treatment. Biomater. Sci..

[10] Mariani F., Serafini M., Gualandi I., Arcangeli D., Decataldo F., Possanzini L., Tessarolo M., Tonelli D., Fraboni B., Scavetta E. (2021). Advanced wound dressing for real-time pH monitoring. ACS Sens..

[11] Jiang Y., Trotsyuk A.A., Niu S., Henn D., Chen K., Shih C.-C., Larson M.R., Mermin-Bunnell A.M., Mittal S., Lai J.-C. (2023). Wireless, closed-loop, smart bandage with integrated sensors and stimulators for advanced wound care and accelerated healing. Nat. Biotechnol..

[12] Chin J.S., Madden L., Chew S.Y., Becker D.L. (2019). Drug therapies and delivery mechanisms to treat perturbed skin wound healing. Adv. Drug Deliv. Rev..

[13] Li M., Xia W., Khoong Y.M., Huang L., Huang X., Liang H., Zhao Y., Mao J., Yu H., Zan T. (2023). Smart and versatile biomaterials for cutaneous wound healing. Biomater. Res..

[14] Nasra S., Pramanik S., Oza V., Kansara K., Kumar A. (2024). Advancements in wound management: Integrating nanotechnology and smart materials for enhanced therapeutic interventions. Discov. Nano.

[15] Deng X., Wu Y., Tang Y., Ge Z., Wang D., Zheng C., Zhao R., Lin W., Wang G. (2024). Microenvironment-responsive smart hydrogels with antibacterial activity and immune regulation for accelerating chronic wound healing. J. Control. Release.

[16] Rani Raju N., Silina E., Stupin V., Manturova N., Chidambaram S.B., Achar R.R. (2022). Multifunctional and Smart Wound Dressings-A Review on Recent Research Advancements in Skin Regenerative Medicine. Pharmaceutics.

[17] Prakashan D., Kaushik A., Gandhi S. (2024). Smart sensors and wound dressings: Artificial intelligence-supported chronic skin monitoring—A review. Chem. Eng. J..

[18] Yu R., Yang Y., He J., Li M., Guo B. (2021). Novel supramolecular self-healing silk fibroin-based hydrogel via host–guest interaction as wound dressing to enhance wound healing. Chem. Eng. J..

[19] Eskilson O., Zattarin E., Berglund L., Oksman K., Hanna K., Rakar J., Sivlér P., Skog M., Rinklake I., Shamasha R. (2023). Nanocellulose composite wound dressings for real-time pH wound monitoring. Mater. Today Bio.

[20] Tang N., Zheng Y., Jiang X., Zhou C., Jin H., Jin K., Wu W., Haick H. (2021). Wearable Sensors and Systems for Wound Healing-Related pH and Temperature Detection. Micromachines.

[21] Wang C., Shirzaei Sani E., Shih C.-D., Lim C.T., Wang J., Armstrong D.G., Gao W. (2024). Wound management materials and technologies from bench to bedside and beyond. Nat. Rev. Mater..

[22] O’Callaghan S., Galvin P., O’Mahony C., Moore Z., Derwin R. (2020). “Smart” wound dressings for advanced wound care: A review. J. Wound Care.

[23] Arafa A.A., Nada A.A., Ibrahim A.Y., Sajkiewicz P., Zahran M.K., Hakeim O.A. (2021). Preparation and characterization of smart therapeutic pH-sensitive wound dressing from red cabbage extract and chitosan hydrogel. Int. J. Biol. Macromol..

[24] Han Z., Yuan M., Liu L., Zhang K., Zhao B., He B., Liang Y., Li F. (2023). pH-Responsive wound dressings: Advances and prospects. Nanoscale Horiz..

[25] Khan M.U.A., Haider S., Raza M.A., Shah S.A., Abd Razak S.I., Kadir M.R.A., Subhan F., Haider A. (2021). Smart and pH-sensitive rGO/Arabinoxylan/chitosan composite for wound dressing: In-vitro drug delivery, antibacterial activity, and biological activities. Int. J. Biol. Macromol..

[26] Alkekhia D., LaRose C., Shukla A. (2022). β-Lactamase-Responsive Hydrogel Drug Delivery Platform for Bacteria-Triggered Cargo Release. ACS Appl. Mater. Interfaces.

[27] Gámez-Herrera E., García-Salinas S., Salido S., Sancho-Albero M., Andreu V., Perez M., Luján L., Irusta S., Arruebo M., Mendoza G. (2020). Drug-eluting wound dressings having sustained release of antimicrobial compounds. Eur. J. Pharm. Biopharm..

[28] Rahimi R., Ochoa M., Parupudi T., Zhao X., Yazdi I.K., Dokmeci M.R., Tamayol A., Khademhosseini A., Ziaie B. (2016). A low-cost flexible pH sensor array for wound assessment. Sens. Actuators B Chem..

[29] Paleček E., Fojta M., Jelen F. (2002). New approaches in the development of DNA sensors: Hybridization and electrochemical detection of DNA and RNA at two different surfaces. Bioelectrochemistry.

[30] Shubham P., Faheem E., Min Z., Roslyn Rivkah I., Bin D., Yubin Z., Yong W., Cunjiang Y. (2022). Wearable electronics for skin wound monitoring and healing. Soft Sci..

[31] Fierheller M., Sibbald R.G. (2010). A clinical investigation into the relationship between increased periwound skin temperature and local wound infection in patients with chronic leg ulcers. Adv. Ski. Wound Care.

[32] Jiang J., Ding J., Wu X., Zeng M., Tian Y., Wu K., Wei D., Sun J., Guo Z., Fan H. (2023). Flexible and temperature-responsive hydrogel dressing for real-time and remote wound healing monitoring. J. Mater. Chem. B.

[33] Zhang K., Lv H., Zheng Y., Yao Y., Li X., Yu J., Ding B. (2021). Nanofibrous hydrogels embedded with phase-change materials: Temperature-responsive dressings for accelerating skin wound healing. Compos. Commun..

[34] Rad I., Esmaeili E., Jahromi B.B. (2024). Application of thermo-responsive polymers as smart biomaterials in wound dressing. Polym. Bull..

[35] Lu S.-H., Samandari M., Li C., Li H., Song D., Zhang Y., Tamayol A., Wang X. (2022). Multimodal sensing and therapeutic systems for wound healing and management: A review. Sens. Actuators Rep..

[36] Ji L., Xiao Y., Xu K., Wu X., Ojo O.W., Diao L., Mequanint K., Zhong W., Zhao P., Xing M. (2025). Smart bandage with multi-sensor system for wound healing and microenvironment monitoring. Chem. Eng. J..

[37] Li X., Zhai W., Tseng Y.-H., Chuang P.-H., Yang Y., She P., Ma X., Tian J., Zhong Z., Liu S. (2025). Wound Dressings Based on Poly (N-Isopropylacrylamide): Design, Performance, and Applications. J. Appl. Polym. Sci..

[38] Ding J., Jiang J., Tian Y., Su B., Zeng M., Wu C., Wei D., Sun J., Luo H., Fan H. (2024). Temperature-Responsive Hydrogel System Integrating Wound Temperature Monitoring and On-demand Drug Release for Sequentially Inflammatory Process Regulation of Wound Healing. ACS Appl. Mater. Interfaces.

[39] Wang X., Shan J., Zhang J., Yang D., Tian G., Dang Y., Ma J. (2025). Synergistic thermoresponsive and photothermal antimicrobial dressing based on bacterial cellulose with surface-grafted PNIPAM and PDA-coated silver nanoparticles for chronic wound treatment. Colloids Surf. A Physicochem. Eng. Asp..

[40] Xiao Y., Xu K., Zhao P., Ji L., Hua C., Jia X., Wu X., Diao L., Zhong W., Lyu G. (2025). Microgels sense wounds’ temperature, pH and glucose. Biomaterials.

[41] da Silva L.P., Reis R.L., Correlo V.M., Marques A.P. (2019). Hydrogel-based strategies to advance therapies for chronic skin wounds. Annu. Rev. Biomed. Eng..

[42] Huang R., Hu J., Qian W., Chen L., Zhang D. (2021). Recent advances in nanotherapeutics for the treatment of burn wounds. Burn. Trauma.

[43] Yang L., Zhang L., Sun D. (2023). Energy harvesting technology based on moisture-responsive actuators. J. Mater. Chem. A.

[44] Yu Y., Zhang F., Liu Y., Leng J. (2025). Smart Polymer fibers: Promising advances in microstructures, stimuli-responsive properties and applications. Adv. Fiber Mater..

[45] Dong L., Zhang W., Ren M., Li Y., Wang Y., Zhou Y., Wu Y., Zhang Z., Di J. (2023). Moisture-Adaptive Contractile Biopolymer-Derived Fibers for Wound Healing Promotion. Small.

[46] Maver U., Gradišnik L., Smrke D.M., Stana Kleinschek K., Maver T. (2019). Impact of growth factors on wound healing in polysaccharide blend thin films. Appl. Surf. Sci..

[47] Tessarolo M., Possanzini L., Gualandi I., Mariani F., Torchia L.D., Arcangeli D., Melandri F., Scavetta E., Fraboni B. (2021). Wireless Textile Moisture Sensor for Wound Care. Front. Phys..

[48] Qin J., Yin L.J., Hao Y.N., Zhong S.L., Zhang D.L., Bi K., Zhang Y.X., Zhao Y., Dang Z.M. (2021). Flexible and stretchable capacitive sensors with different microstructures. Adv. Mater..

[49] Mishra R.B., El-Atab N., Hussain A.M., Hussain M.M. (2021). Recent progress on flexible capacitive pressure sensors: From design and materials to applications. Adv. Mater. Technol..

[50] Ding S., Jin X., Guo J., Kou B., Chai M., Dou S., Jin G., Zhang H., Zhao X., Ma J. (2025). A biomimetic asymmetric structured intelligent wound dressing with dual-modality humidity-pressure sensing for non-invasive and real-time wound healing monitoring. Adv. Fiber Mater..

[51] Li D., Fei X., Xu L., Wang Y., Tian J., Li Y. (2022). Pressure-sensitive antibacterial hydrogel dressing for wound monitoring in bed ridden patients. J. Colloid. Interface Sci..

[52] Orgill D.P., Bayer L.R. (2013). Negative pressure wound therapy: Past, present and future. Int. Wound J..

[53] Jin S., Newton M.A.A., Cheng H., Zhang Q., Gao W., Zheng Y., Lu Z., Dai Z., Zhu J. (2023). Progress of hydrogel dressings with wound monitoring and treatment functions. Gels.

[54] Zhang J., Luo Q., Hu Q., Zhang T., Shi J., Kong L., Fu D., Yang C., Zhang Z. (2023). An injectable bioactive dressing based on platelet-rich plasma and nanoclay: Sustained release of deferoxamine to accelerate chronic wound healing. Acta Pharm. Sin. B.

[55] Lee Y., Kim H., Kim Y., Noh S., Chun B., Kim J., Park C., Choi M., Park K., Lee J. (2021). A multifunctional electronic suture for continuous strain monitoring and on-demand drug release. Nanoscale.

[56] Singh B., Kim K., Park M.-H. (2021). On-demand drug delivery systems using nanofibers. Nanomaterials.

[57] Zeng Z., Guo C., Lu D., Geng Z., Pei D., Yu S. (2022). Polyphenol–Metal Functionalized Hydrogel Dressing with Sustained Release, Antibacterial, and Antioxidant Properties for the Potential Treatment of Chronic Wounds. Macromol. Mater. Eng..

[58] Hu X., Zhang H., Lin Z.-T., Chen Y., Cheng H., Zhong B.-H., Li Y., Liao X.-P., Sun J., Wu M.X. (2025). A remotely triggered phase change hydrogel dressing for controlled release and promoting healing of infected wounds. Chem. Eng. J..

[59] Abedin-Do A., Zhang Z., Douville Y., Méthot M., Rouabhia M. (2021). Effect of Electrical Stimulation on Diabetic Human Skin Fibroblast Growth and the Secretion of Cytokines and Growth Factors Involved in Wound Healing. Biology.

[60] Uberoi A., McCready-Vangi A., Grice E.A. (2024). The wound microbiota: Microbial mechanisms of impaired wound healing and infection. Nat. Rev. Microbiol..

[61] Aycan D., Selmi B., Kelel E., Yildirim T., Alemdar N. (2019). Conductive polymeric film loaded with ibuprofen as a wound dressing material. Eur. Polym. J..

[62] Khan M.U.A., Stojanovic G.M., Hassan R., Anand T.J.S., Al-Ejji M., Hasan A. (2023). Role of graphene oxide in bacterial cellulose− gelatin hydrogels for wound dressing applications. ACS Omega.

[63] Vijayakumar G., Kim H.J., Rangarajulu S.K. (2023). In Vitro Antibacterial and Wound Healing Activities Evoked by Silver Nanoparticles Synthesized through Probiotic Bacteria. Antibiotics.

[64] Wei Z., Xu T., Wang C., Liu S., Zhang W., Sun J., Yu H., Shi H., Song Y. (2024). A hydrogel-functionalized silver nanocluster for bacterial-infected wound healing. Nanoscale.

[65] Ojingwa J.C., Isseroff R.R. (2003). Electrical stimulation of wound healing. J. Investig. Dermatol..

[66] Liang Y., Qiao L., Qiao B., Guo B. (2023). Conductive hydrogels for tissue repair. Chem. Sci..

[67] Shan M., Chen X., Zhang X., Zhang S., Zhang L., Chen J., Wang X., Liu X. (2024). Injectable Conductive Hydrogel with Self-Healing, Motion Monitoring, and Bacteria Theranostics for Bioelectronic Wound Dressing. Adv. Healthc. Mater..

[68] Yu R., Zhang H., Guo B. (2021). Conductive Biomaterials as Bioactive Wound Dressing for Wound Healing and Skin Tissue Engineering. Nano-Micro Lett..

[69] Wang C., Jiang X., Kim H.-J., Zhang S., Zhou X., Chen Y., Ling H., Xue Y., Chen Z., Qu M. (2022). Flexible patch with printable and antibacterial conductive hydrogel electrodes for accelerated wound healing. Biomaterials.

[70] Deng P., Chen F., Zhang H., Chen Y., Zhou J. (2021). Conductive, self-healing, adhesive, and antibacterial hydrogels based on lignin/cellulose for rapid MRSA-infected wound repairing. ACS Appl. Mater. Interfaces.

[71] Liu Q., Wang C., Cheng M., Hu L., Zhang Z., Sun Q., Wang S., Fan Y., Pan P., Chen J. (2024). Self-Healing Conductive Hydrogels with Dynamic Dual Network Structure Accelerate Infected Wound Healing via Photothermal Antimicrobial and Regulating Inflammatory Response. ACS Appl. Mater. Interfaces.

[72] Wu Z., Cheng T., Wang Z.L. (2020). Self-powered sensors and systems based on nanogenerators. Sensors.

[73] Li S., Wang L., Zheng W., Yang G., Jiang X. (2020). Rapid Fabrication of Self-Healing, Conductive, and Injectable Gel as Dressings for Healing Wounds in Stretchable Parts of the Body. Adv. Funct. Mater..

[74] Li W., Liu Z., Tan X., Yang N., Liang Y., Feng D., Li H., Yuan R., Zhang Q., Liu L. (2024). All-in-one self-powered microneedle device for accelerating infected diabetic wound repair. Adv. Healthc. Mater..

[75] Li W., Tan X., Liang Y., Chen N., Feng D., Tao Y., Liu T., Wu X., Lu H., Liu L. (2025). Multifunctional microneedle patch synergized with electrical stimulation to regulate microenvironment for enhanced healing of infected diabetic wounds. Chem. Eng. J..

[76] Cao W., Peng S., Yao Y., Xie J., Li S., Tu C., Gao C. (2022). A nanofibrous membrane loaded with doxycycline and printed with conductive hydrogel strips promotes diabetic wound healing in vivo. Acta Biomater..

[77] Palani N., Mendonce K.C., Syed Altaf R.R., Mohan A., Surya P., P M., Radhakrishnan K., Subramaniyan V., Rajadesingu S. (2025). Next-generation smart wound dressings: AI integration, biosensors, and electrospun nanofibers for chronic wound therapy. J. Biomater. Sci. Polym. Ed..

[78] Ali M.K., Pearson-Stuttard J., Selvin E., Gregg E.W. (2022). Interpreting global trends in type 2 diabetes complications and mortality. Diabetologia.

[79] Mishra A., Kushare A., Gupta M.N., Ambre P. (2024). Advanced dressings for chronic wound management. ACS Appl. Bio Mater..

[80] Arcangeli D., Gualandi I., Mariani F., Tessarolo M., Ceccardi F., Decataldo F., Melandri F., Tonelli D., Fraboni B., Scavetta E. (2023). Smart Bandaid Integrated with Fully Textile OECT for Uric Acid Real-Time Monitoring in Wound Exudate. ACS Sens..

[81] Chen M., Ren X., Dong L., Li X., Cheng H. (2021). Preparation of dynamic covalently crosslinking keratin hydrogels based on thiol/disulfide bonds exchange strategy. Int. J. Biol. Macromol..

[82] Xiang T., Guo Q., Jia L., Yin T., Huang W., Zhang X., Zhou S. (2024). Multifunctional hydrogels for the healing of diabetic wounds. Adv. Healthc. Mater..

[83] Kampangsat S., Kajornprai T., Tangjatuporn W., Suppakarn N., Trongsatitkul T. (2024). Enhancing Tensile Modulus of Polyurethane-Based Shape Memory Polymers for Wound Closure Applications through the Addition of Palm Oil. Polymers.

[84] Mao Q., Hoffmann O., Yu K., Lu F., Lan G., Dai F., Shang S., Xie R. (2020). Self-contracting oxidized starch/gelatin hydrogel for noninvasive wound closure and wound healing. Mater. Des..

[85] Petryk N.M., Thai N.L.B., Saldanha L.V., Sutherland S.T., Monroe M.B. (2025). Bioactive Polyurethane Shape Memory Polymer Foam Dressings with Enhanced Blood and Cell Interactions for Improved Wound Healing. ACS Appl. Mater. Interfaces.

[86] Wang X., Zhong B., Lou Z., Han W., Wang L. (2024). The advancement of intelligent dressings for monitoring chronic wound infections. Chem. Eng. J..

[87] Xu P., Kankala R.K., Wang S., Chen A. (2024). Decellularized extracellular matrix-based composite scaffolds for tissue engineering and regenerative medicine. Regen. Biomater..

[88] Li M., Zhang Y., Lian L., Liu K., Lu M., Chen Y., Zhang L., Zhang X., Wan P. (2022). Flexible accelerated-wound-healing antibacterial MXene-based epidermic sensor for intelligent wearable human-machine interaction. Adv. Funct. Mater..

[89] Kalasin S., Sangnuang P., Surareungchai W. (2022). Intelligent wearable sensors interconnected with advanced wound dressing bandages for contactless chronic skin monitoring: Artificial intelligence for predicting tissue regeneration. Anal. Chem..

[90] Zou Y., Wang P., Zhang A., Qin Z., Li Y., Xianyu Y., Zhang H. (2022). Covalent organic framework-incorporated nanofibrous membrane as an intelligent platform for wound dressing. ACS Appl. Mater. Interfaces.

[91] Shen Z., Zhang C., Wang T., Xu J. (2023). Advances in functional hydrogel wound dressings: A review. Polymers.

[92] Zeng Q., Qi X., Shi G., Zhang M., Haick H. (2022). Wound dressing: From nanomaterials to diagnostic dressings and healing evaluations. ACS Nano.

[93] Deng D., Liang L., Su K., Gu H., Wang X., Wang Y., Shang X., Huang W., Chen H., Wu X. (2025). Smart hydrogel dressing for machine learning-enabled visual monitoring and promote diabetic wound healing. Nano Today.

[94] King S.W., Abouharb A., Doggett T., Taufiqurrakhman M., Palan J., Freear B., Pandit H., van Duren B.H. (2024). A Scoping Review of ‘Smart’ Dressings for Diagnosing Surgical Site Infection: A Focus on Arthroplasty. Bioengineering.

[95] Wang S., Wu W.Y., Yeo J.C.C., Soo X.Y.D., Thitsartarn W., Liu S., Tan B.H., Suwardi A., Li Z., Zhu Q. (2023). Responsive hydrogel dressings for intelligent wound management. BMEMat.

[96] Ge Z., Guo W., Tao Y., Sun H., Meng X., Cao L., Zhang S., Liu W., Akhtar M.L., Li Y. (2023). Wireless and closed-loop smart dressing for exudate management and on-demand treatment of chronic wounds. Adv. Mater..

